# Discrimination between uterine serous papillary carcinomas and ovarian serous papillary tumours by gene expression profiling

**DOI:** 10.1038/sj.bjc.6601791

**Published:** 2004-04-27

**Authors:** A D Santin, F Zhan, S Bellone, M Palmieri, S Cane, M Gokden, J J Roman, T J O'Brien, E Tian, M J Cannon, J Shaughnessy, S Pecorelli

**Affiliations:** 1 Department of Obstetrics & Gynecology, Division of Gynecologic Oncology, University of Arkansas for Medical Sciences, Little Rock, AR, USA; 2 Department of Medicine, Myeloma Institute for Research and Therapy, University of Arkansas for Medical Sciences, Little Rock, AR, USA; 3 Department of Pathology, University of Arkansas for Medical Sciences, Little Rock, AR, USA; 4 Department of Microbiology & Immunology, University of Arkansas for Medical Sciences, Little Rock, AR, USA; 5 Division of Gynecologic Oncology, University of Brescia, Brescia, Italy

**Keywords:** serous papillary uterine cancer, serous papillary ovarian cancer, gene expression profiling, Her-2/neu, herceptin

## Abstract

High-grade ovarian serous papillary cancer (OSPC) and uterine serous papillary carcinoma (USPC) represent two histologically similar malignancies characterised by markedly different biological behavior and response to chemotherapy. Understanding the molecular basis of these differences may significantly refine differential diagnosis and management, and may lead to the development of novel, more specific and more effective treatment modalities for OSPC and USPC. We used an oligonucleotide microarray with probe sets complementary to >10 000 human genes to determine whether patterns of gene expression may differentiate OSPC from USPC. Hierarchical cluster analysis of gene expression in OSPC and USPC identified 116 genes that exhibited >two-fold differences (*P*<0.05) and that readily distinguished OSPC from USPC. Plasminogen activator inhibitor (PAI-2) was the most highly overexpressed gene in OSPC when compared to USPC, while c-erbB2 was the most strikingly overexpressed gene in USPC when compared to OSPC. Overexpression of the c-erbB2 gene and its expression product (i.e., HER-2/neu receptor) was validated by quantitative RT–PCR as well as by flow cytometry on primary USPC and OSPC, respectively. Immunohistochemical staining of serous tumour samples from which primary OSPC and USPC cultures were derived as well as from an independent set of 20 clinical tissue samples (i.e., 10 OSPC and 10 USPC) further confirmed HER-2/neu as a novel molecular diagnostic and therapeutic marker for USPC. Gene expression fingerprints have the potential to predict the anatomical site of tumour origin and readily identify the biologically more aggressive USPC from OSPC. A therapeutic strategy targeting HER-2/neu may be beneficial in patients harbouring chemotherapy-resistant USPC.

Ovarian serous papillary cancer (OSPC) represents the most common histological type of ovarian carcinoma, the fourth leading cause of cancer-related death in women in the United States (Jemal *et al*, 2003). Endometrial cancer is the most frequent cancer of the female genital tract with endometrioid (type 1) and serous papillary (type 2) being the most common cell types (Deligdisch and Holinka, 1987; Jemal *et al*, 2003). Histologically indistinguishable to high-grade serous ovarian carcinoma (Carcangiu and Chambers, 1992; Sherman *et al*, 1992), uterine serous papillary cancer (USPC) has a propensity for early intraabdominal, lymphatic and distant metastatic spread even at presentation (Carcangiu and Chambers, 1992; Goff *et al*, 1994; Nicklin and Copeland, 1996) and is characterised by a highly aggressive biological behavior (Deligdisch and Holinka, 1987; Carcangiu and Chambers, 1992; Sherman *et al*, 1992; Goff *et al*, 1994; Nicklin and Copeland, 1996). Unlike OSPC, however, which is responsive to first-line combined cisplatinum-based chemotherapy in 70–80% of the cases (Kalil and McGuire, 2002), USPC is a chemotherapy-resistant disease from outset, with responses to cytostatic agents in the order of 20% and of short duration (Levenback *et al*, 1992; Carcangiu and Chambers, 1995; Nicklin and Copeland, 1996).

Gene expression fingerprints representing large numbers of genes have the potential to allow precise and accurate grouping of tumours endowed with similar phenotype (Giordano *et al*, 2001; Sorlie *et al*, 2001; Rosenwald *et al*, 2002; Zhan *et al*, 2002). Gene microarrays may identify cancers endowed with a more aggressive biologic behaviour (i.e., rapidly metastatic tumours) that are unresponsive to standard adjuvant therapies and may thus allow improved prediction of response and clinical outcome. Consistent with this view, in large B-cell lymphomas and breast carcinomas, gene expression profiles have been shown to identify patients who are unlikely to be cured by conventional therapy (Sorlie *et al*, 2001; Rosenwald *et al*, 2002). In ovarian carcinoma, cDNA microarray technology has recently been used to identify numerous genes differentially expressed in normal and tumour-derived ovarian epithelial cells (Ismail *et al*, 2000; Hough *et al*, 2001; Welsh *et al*, 2001; Schwartz *et al*, 2002). Interestingly, several of the most upregulated genes encode surface or secreted proteins, such as Kop, SLPI and claudin-3, making these products attractive candidate biomarkers (Ismail *et al*, 2000; Hough *et al*, 2001; Welsh *et al*, 2001; Schwartz *et al*, 2002). In contrast, very little is known about the possible genetic diversity between OSPC and USPC, two histologically similar serous carcinomas characterised by a dramatically different biological behavior and response to chemotherapy.

In this study, oligonucleotide microarrays were used to profile and compare gene expression patterns in 11 primary cultures of OSPC and USPC. We report that mRNA fingerprints readily distinguish the more biologically aggressive and chemotherapy resistant USPC from OSPC. Of interest, OSPC2, a primary OSPC with mixed clear cell features (a variant of ovarian cancer also characterised with a particularly unfavourable prognosis), clustered with USPC. Plasminogen activator inhibitor (PAI-2) was the gene most highly upregulated in OSPC relative to USPC, while the c-erbB2 gene product (HER-2/neu) was strikingly overexpressed in USPC relative to OSPC and may therefore represent a novel diagnostic and therapeutic marker for this highly aggressive subset of endometrial tumours.

## MATERIALS AND METHODS

### Establishment of OSPC and USPC primary cell lines

In all, 11 primary serous papillary cell lines (six OSPC and five USPC) were established after sterile processing of the tumour samples from surgical biopsies as described for ovarian and uterine carcinoma specimens (Santin *et al*, 2000, 2002a, 2002b). All tumour samples were obtained with appropriate consent according to IRB guidelines. Tumours were staged according to the FIGO operative staging system. Total abdominal hysterectomy and regional lymph node sampling for invasive USPC were performed in all cases. Radical tumour debulking, including a total abdominal hysterectomy and omentectomy, was performed in all ovarian carcinoma patients. No patient received chemotherapy before surgical therapy. The patient characteristics are described in Table 1

**Table 1:** Characteristics of the patients

**Patient**	**Age**	**Race**	**Stage**	**Chemotherapy regimen**
USPC 1	66	Afro-American	IV B	TAX+CARB
USPC 2	77	White	III C	TAX+CARB
USPC 3	61	Afro-American	III C	TAX+CARB
USPC 4	62	Afro-American	III C	TAX+CARB
USPC 5	63	Afro-American	III C	TAX+CARB
				
OSPC 1	42	White	III C	TAX+CIS
OSPC 2	43	White	III C	TAX+CARB
OSPC 3	34	White	III C	TAX+CARB
OSPC 4	51	White	III C	TAX+CARB
OSPC 5	59	Afro-American	III B	TAX+CARB
OSPC 6	52	White	III C	TAX+CARB

. The epithelial nature and the purity of USPC and OSPC cultures was verified by immunohistochemical staining and flow cytometric analysis with antibodies against cytokeratin as described (Ismail *et al*, 2000; Santin *et al*, 2000, 2002a, 2002b). Only primary cultures which had at least 90% viability and contained >99% tumour cells were used for total RNA extraction.

### RNA purification, microarray hybridisation and analysis

RNA purification, cDNA synthesis, cRNA preparation and hybridisation to the Affymetrix Human U95Av2 GeneChip microarray were performed according to the manufacturer's protocols and as reported (Zhan *et al*, 2002).

### Data processing

All data used in our analyses were derived from Affymetrix 5.0 software. GeneChip 5.0 output files are given as a signal that represents the difference between the intensities of the sequence-specific perfect match probe set and the mismatch probe set, or as a detection of present, marginal, or absent signals as determined by the GeneChip 5.0 algorithm. Gene arrays were scaled to an average signal of 1500 and then analysed independently. Signal calls were transformed by the log base 2 and each sample was normalised to give a mean of 0 and variance of 1.

### Gene expression data analysis

Statistical analyses of the data were performed with the software packages SPSS10.0. (SPSS, Chicago, IL, USA) and the significance analysis of microarrays (SAM) method (Tusher *et al*, 2001). Genes were selected for analysis based on detection and fold change. In each comparison, genes having ‘present’ detection calls in more than half of the samples in the overexpressed gene group were retained for statistical analysis if they showed >two-fold change between groups. Retained genes were subjected to SAM to establish a false discovery rate (FDR), then further filtered via the Wilcoxon rank-sum (WRS) test at *α*=0.05. The FDR obtained from the initial SAM analysis was assumed to characterise genes found significant via WRS.

### Gene cluster/treeview

**Figure 1:**
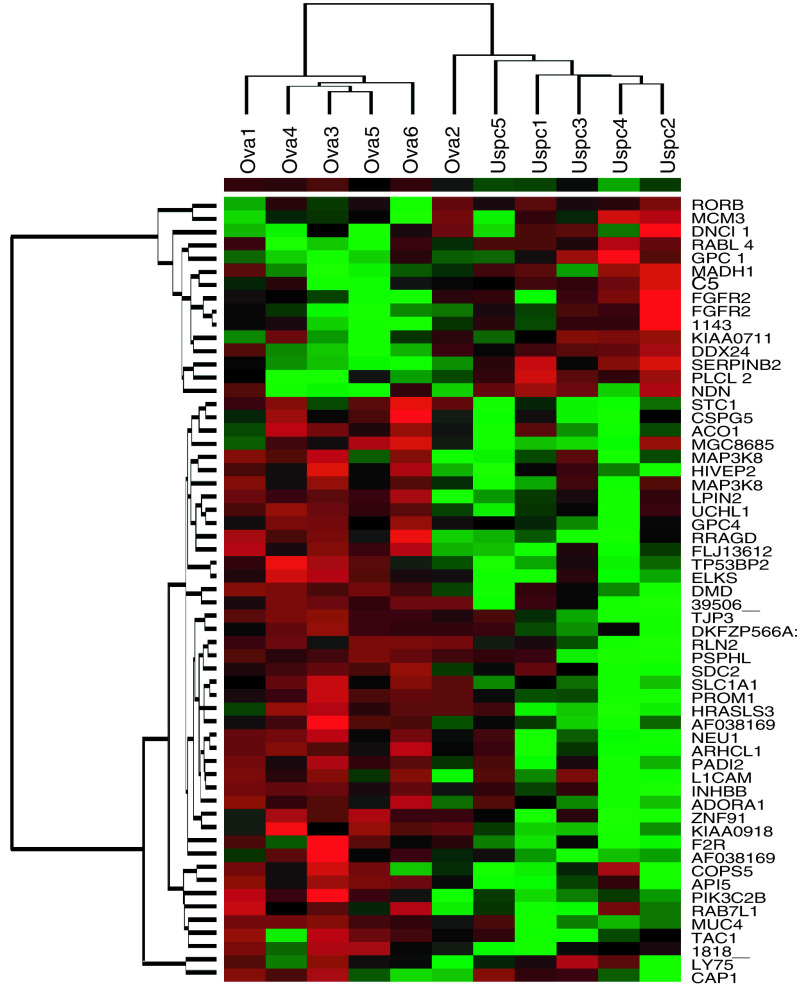
Molecular profile of 11 primary OSPC and USPC cell lines. Hierarchical clustering of 59 genes with differential expression between six OSPC and five USPC groups (*P*<0.05) using a two-fold threshold. The cluster is colour coded using *red* for upregulation, *green* for downregulation and *black* for median expression. Agglomerative clustering of genes was illustrated with dendrograms. The symbol for each gene corresponding to the oligonucleotide spotted on the array is shown.

The hierarchical clustering of average-linkage method with the centred correlation metric was used (Eisen *et al*, 1998). The dendrogram was constructed with a subset of genes from 12 588 probe sets present on the microarray, whose expression levels vary the most among the 11 samples, and thus most informative. For the hierarchical clustering shown in Figures 1

and 2

**Figure 2:**
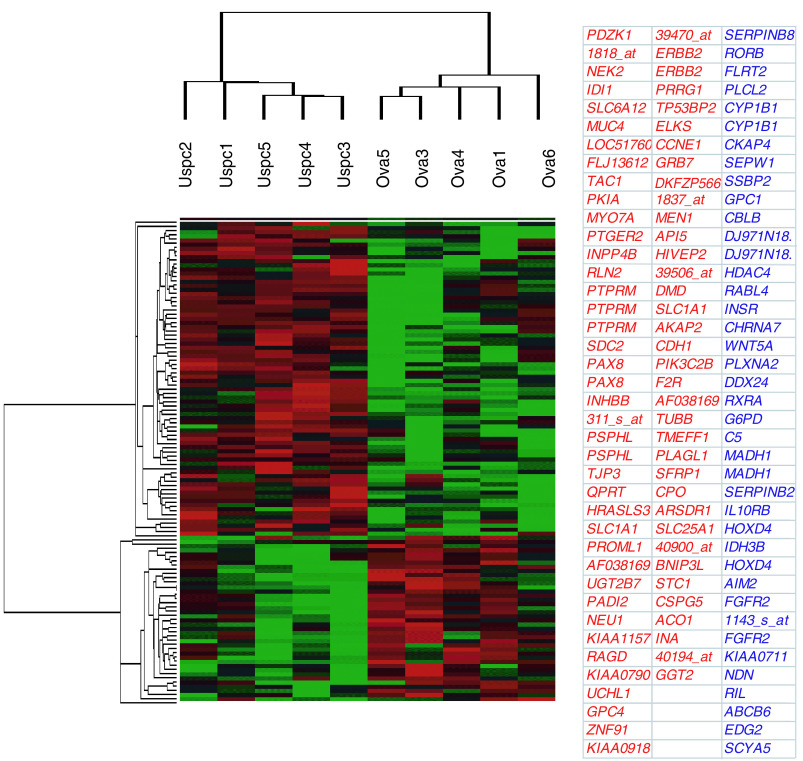
Molecular profile of primary OSPC and USPC cell lines. Hierarchical clustering of 116 genes with differential expression between five OSPC and five USPC groups (*P*<0.05) using a two-fold threshold. The cluster is colour coded using *red* for upregulation, *green* for downregulation and *black* for median expression. Agglomerative clustering of genes was illustrated with dendrograms. The symbol for each gene corresponding to the oligonucleotide spotted on the array is shown. USPC upregulated genes are shown in red ink while OSPC upregulated genes are shown in blue ink.

, only genes significantly expressed and whose average change in expression level was at least two-fold were chosen. The expression value of each selected gene was re-normalized to have a mean of zero.

### Quantitative real-time PCR

q-RT – PCR was performed with an ABI Prism 7000 Sequence Analyzer using the manufacturer's recommended protocol (Applied Biosystems, Foster City, CA, USA) to validate differential expression of selected genes in samples from six representative primary tumour cell lines (three OSPC and three USPC). Each reaction was run in triplicate. The comparative threshold cycle (*C*_T_) method was used for the calculation of amplification fold as specified by the manufacturer. Briefly, 5 *μ*g of total RNA from each sample was reverse transcribed using SuperScript II Rnase H Reverse Transcriptase (Invitrogen, Carlsbad, CA, USA). A value of 10 *μ*l of reverse-transcribed RNA samples (from 500 *μ*l of total volume) was amplified by using the TaqMan Universal PCR Master Mix (Applied Biosystems) to produce PCR products specific for *PAI-2* and *c-erbB2.* Primers specific for 18s ribosomal RNA and empirically determined ratios of 18 s competimers (Applied Biosystems) were used to control for the amounts of cDNA generated from each sample. Sequences for primers and probes are available on request. Differences among OSPC and USPC in the q-RT – PCR expression data were tested using the Kruskal–Wallis nonparametric test. Pearson's product – moment correlations were used to estimate the degree of association between the microarray and q-RT – PCR data.

### Flow cytometry

To validate microarray data on primary OSPC and USPC cell lines at the protein level, HER-2/neu receptor expression was evaluated by flow cytometry. The HER-2/neu MAb Herceptin (Genentech, San Francisco, CA, USA) was used as the primary antibody. FITC-conjugated goat anti-human F(ab)^2^ immunoglobulin was used as a secondary reagent (BioSource International, Camarillo, CA, USA). Analysis was conducted with a FACScan, utilising Cell Quest software (Becton Dickinson).

### HER2/neu immunostaining of formalin-fixed tumour tissues

To evaluate whether the differential HER2/Neu receptor expression detected by flow cytometry on primary OSPC and USPC cell lines was comparable to the expression of HER-2/neu receptor of uncultured OSPC and USPC from which the primary cell lines were derived, protein expression was evaluated by immunohistochemical staining on formalin-fixed tumour tissue. In addition, to further confirm transcriptional profiling results, the HER2/neu marker was also evaluated by immunohistochemistry in a second independent set of 20 clinical tissue samples (i.e., 10 OSPC and 10 USPC) obtained from patients harbouring advanced stage disease (i.e., stages III and IV). Study blocks were selected after histopathologic review by a surgical pathologist. The intensity of staining was graded as 0 (staining not greater than negative control), 1+ (light staining), 2+ (moderate staining) or 3+ (heavy staining).

## RESULTS

### Gene expression profiles distinguish OSPC from USPC and identify differentially expressed genes

Flash frozen biopsies from ovarian and uterine tumour tissue are known to contain significant numbers of contaminant stromal cells as well as a variety of host-derived immune cells (e. g., monocytes, dendritic cells, lymphocytes). Short-term primary OSPC and USPC cell cultures, minimising the risk of a selection bias inherent in any long-term *in vitro* growth, provide an opportunity to study differential gene expression between relatively pure populations of tumour cells. Comprehensive gene expression profiles of six primary OSPC and five primary USPC cell lines were generated using high-density oligonucleotide arrays with 12 588 probe sets, which in total interrogated some 10 000 genes. In total, 165 genes were differentially expressed between OSPC and USPC (WRS test, *P*<0.05). Figure 1 shows the cluster analysis performed on hybridisation intensity values for 59 gene segments whose average difference in expression level was at least two-fold. Two major branches on the dendrogram were identified. All five USPC were grouped together in the rightmost columns. Similarly, in the leftmost columns five pure OSPC were found to cluster tightly together. Of interest, OSPC2, a serous papillary tumour with mixed clear cell features (i.e., a biologically aggressive variant of ovarian cancer characterised by a poor prognosis) clustered on a sub-branch with USPC (Figure 1). Figure 2 shows the cluster analysis on hybridisation intensity values for each gene in 10 primary cultures of OSPC and USPC showing a single type of differentiation. There were 484 genes showing >two-fold change along with ‘present’ detection calls in more than half the samples in the overexpressed group. Of these, 316 were found significant by SAM, with a median FDR of 17.4% and a 90th percentile FDR of 22.7%. Of the 484 aforementioned genes, 116 yielded *P*<0.05 via WRS, and all 116 were among the genes found significant by SAM. Thus, we can say with 90% confidence that the FDR among genes found significant via WRS is no higher than 22.7%. The new dendrogram shown in Figure 2 depicts a marked separation in the expression profiles of the two groups of serous papillary tumours. The tight clustering of pure OSPC from USPC was driven by two distinct profiles of gene expression. The first was represented by a group of 40 genes that were highly expressed in OSPC and underexpressed in USPC (Table 2

**Table 2:** Upregulated genes expressed at least two fold higher in OSPC compared with USPC

**Probe set name**	**Gene symbol**	**Map location**	**p of WRS**	**Ratio Ov/Ut**
37185_at	SERPINB2	18q21.3	0.00902344	21.2101742
40478_at	DJ971N18.2	20p12	0.0162936	7.391447995
38837_at	DJ971N18.2	20p12	0.047201768	6.933671714
34439_at	AIM2	1q22	0.00902344	6.689727463
36073_at	NDN	15q11.2-q12	0.028280124	6.460327167
859_at	CYP1B1	2p21	0.047201768	4.642443935
40387_at	EDG2	9q32	0.047201768	4.612620508
1669_at	WNT5A	3p21-p14	0.028280124	4.35214472
1363_at	FGFR2	10q26	0.0162936	3.958060853
1143_s_at			0.028280124	3.948020982
37816_at	C5	9q32-q34	0.0162936	3.945622621
40071_at	CYP1B1	2p21	0.047201768	3.826875845
38294_at	HOXD4	2q31-q37	0.028280124	3.804399853
33162_at	INSR	19p13.3-p13.2	0.047201768	3.772
34853_at	FLRT2	14q24-q32	0.047201768	3.471204819
40395_at	PLXNA2	1q32.1	0.028280124	3.371729137
39805_at	ABCB6	2q36	0.047201768	3.369062784
41796_at	PLCL2	3p24.3	0.00902344	3.280007364
1403_s_at	SCYA5	17q11.2-q12	0.047201768	3.158368265
33929_at	GPC1	2q35-q37	0.028280124	3.15594993
39566_at	CHRNA7	15q14	0.047201768	3.14079953
34354_at	FGFR2	10q26	0.047201768	2.928346342
444_g_at	HOXD4	2q31-q37	0.047201768	2.892672123
38042_at	G6PD	Xq28	0.047201768	2.813117012
36077_at	RABL4	22q13.1	0.028280124	2.720984156
36453_at	KIAA0711	8p23.3	0.047201768	2.688792044
32668_at	SSBP2	5q14.1	0.047201768	2.663148439
32610_at	RIL	5q31.1	0.047201768	2.55031145
514_at	CBLB	3q13.12	0.028280124	2.511893491
40112_at	IDH3B	20p13	0.028280124	2.294973901
38271_at	HDAC4	2q37.2	0.028280124	2.245891142
1325_at	MADH1	4q28	0.047201768	2.228503651
32381_at	RORB	9q22	0.028280124	2.205852674
32800_at	RXRA	9q34.3	0.047201768	2.168594631
36312_at	SERPINB8	18q21.3	0.047201768	2.110497544
40142_at	DDX24	14q32	0.0162936	2.109997452
33227_at	IL10RB	21q22.11	0.047201768	2.082986437
32529_at	CKAP4	12q23.3	0.047201768	2.04858844
37280_at	MADH1	4q28	0.028280124	2.044781456
39709_at	SEPW1	19q13.3	0.028280124	2.017195806

). Many genes shown previously to be involved in ovarian carcinogenesis are present on these lists, providing a degree of validity to our array analysis. Included in this group of genes are *plasminogen activator inhibitor-2* (*PAI-2*), *fibroblast growth factor receptor-2 (FGFR2), glypican 1 (GPC1), lysophosphatidic acid receptor (EDG2), phospholipase C (PLCL2), glucose-6-phosphate dehydrogenase (G6PD)* and *insulin receptor (IGF1)* (Table 2). The second profile was represented by 76 genes that were highly expressed in USPC and underexpressed in OSPC (Table 3

**Table 3:** Upregulated genes expressed at least two-fold higher in USPC compared with OSPC

**Probe set name**	**Gene symbol**	**Map location**	**p of WRS**	**Ratio Ut/Ov**
1802_s_at	ERBB2	17q11.2-q12	0.028280124	17.39166248
39470_at			0.00902344	14.13960749
41470_at	PROML1	4p15.33	0.00902344	11.00274366
32521_at	SFRP1	8p12-p11.1	0.047201768	10.49619245
33218_at	ERBB2	17q11.2-q12	0.0162936	9.009761458
41354_at	STC1	8p21-p11.2	0.0162936	7.780569927
41700_at	F2R	5q13	0.028280124	7.299013748
38207_at	MEN1	11q13	0.028280124	6.578419265
36254_at	TAC1	7q21-q22	0.047201768	6.292979547
38268_at	SLC1A1	9p24	0.0162936	5.506571087
33576_at	KIAA0918	13q31.1	0.0162936	5.478319783
37883_i_at	AF038169	2q22.1	0.0162936	5.06566416
35704_at	HRASLS3	11q13.1	0.028280124	4.596441783
38267_at	SLC1A1	9p24	0.028280124	4.488128886
41376_i_at	UGT2B7	4q13	0.047201768	4.418941048
828_at	PTGER2	14q22	0.028280124	4.338041431
39506_at			0.028280124	4.313685637
1680_at	GRB7	17q12	0.047201768	4.262623744
38545_at	INHBB	2cen-q13	0.028280124	4.198823428
40679_at	SLC6A12	12p13	0.047201768	3.956969879
35912_at	MUC4	3q29	0.028280124	3.94095027
39966_at	CSPG5	3p21.3	0.047201768	3.918103678
32027_at	PDZK1	1q21	0.047201768	3.91484375
31732_at	RLN2	9p24.1	0.0162936	3.913095715
36202_at	PKIA	8q21.11	0.047201768	3.89984472
37978_at	QPRT	16q13	0.0162936	3.845374532
994_at	PTPRM	18p11.2	0.047201768	3.812843137
37208_at	PSPHL	7q11.2	0.028280124	3.654717567
37884_f_at	AF038169	2q22.1	0.028280124	3.593346825
995_g_at	PTPRM	18p11.2	0.028280124	3.555706062
35985_at	AKAP2	9q31-q33	0.028280124	3.319448607
32963_s_at	RAGD	6q15-q16	0.00902344	3.280777993
33358_at	KIAA1157	12q13.13	0.0162936	3.250881457
311_s_at			0.0162936	3.138465417
35674_at	PADI2	1p35.2-p35.1	0.047201768	3.100307522
2021_s_at	CCNE1	19q12	0.028280124	3.081090355
32893_s_at	GGT2	22q11.23	0.047201768	3.055014721
36869_at	PAX8	2q12-q14	0.047201768	3.050015496
36508_at	GPC4	Xq26.1	0.0162936	2.887073572
39901_at	MYO7A	11q13.5	0.028280124	2.885983264
35148_at	TJP3	19p13.3	0.028280124	2.879832572
31892_at	PTPRM	18p11.2	0.047201768	2.844557651
36990_at	UCHL1	4p14	0.0162936	2.833524684
37209_g_at	PSPHL	7q11.2	0.047201768	2.780479031
38168_at	INPP4B	4q31.1	0.00902344	2.645321215
36943_r_at	PLAGL1	6q24-q25	0.0162936	2.57527834
37258_at	TMEFF1	9q31	0.047201768	2.55946924
36985_at	IDI1	10p15.3	0.047201768	2.538587569
39075_at	NEU1	6p21.3	0.0162936	2.521110072
40488_at	DMD	Xp21.2	0.00902344	2.507697552
39332_at	TUBB	6p21.3	0.047201768	2.504487188
39757_at	SDC2	8q22-q23	0.047201768	2.452025072
933_f_at	ZNF91	19p13.1-p12	0.028280124	2.445525292
37210_at	INA	10q25.1	0.047201768	2.387532735
1860_at	TP53BP2	1q42.1	0.0162936	2.356857655
37869_at	ELKS	12p13.3	0.028280124	2.356300578
33878_at	FLJ13612	2q36.1	0.0162936	2.319659881
35143_at	DKFZP566A1524		0.047201768	2.312331476
38997_at	SLC25A1	22q11.21	0.00902344	2.304275318
40077_at	ACO1	9p22-p13	0.028280124	2.297124855
36261_at	LOC51760	16p13.13	0.028280124	2.252602915
39436_at	BNIP3L	8p21	0.047201768	2.236567978
977_s_at	CDH1	16q22.1	0.00902344	2.212331718
36175_s_at	HIVEP2	6q23-q24	0.047201768	2.206300362
41269_r_at	API5	11p12-q12	0.0162936	2.189353711
1837_at			0.047201768	2.180124558
1818_at			0.047201768	2.177494716
366_s_at	NEK2	1q32.2-q41	0.047201768	2.157771457
40900_at			0.028280124	2.151464435
40194_at			0.028280124	2.133081444
41172_at	ARSDR1	14q23.3	0.0162936	2.113388456
37999_at	CPO	3q12	0.028280124	2.100322069
35978_at	PRRG1	Xp21.1	0.028280124	2.05552932
121_at	PAX8	2q12-q14	0.028280124	2.028946437
41715_at	PIK3C2B	1q32	0.00902344	2.024856688
41644_at	KIAA0790	6q24.3	0.047201768	2.004743183

). Included in this group of genes are *epidermal growth factor type 2 receptor (c-erbB2), inhibin (INHBB), multiple endocrine neoplasia I (MEN1), growth factor receptor-bound protein 7 (GRB7), BCL2, E-cadherin (CDH1)* and *syndecan (SDC2)* (Table 3). Importantly, *c-erbB2* gene was the most highly differentially expressed gene in USPC when compared to OSPC (Table 3, Table 4

**Table 4:** Differentially expressed genes in USPC and OSPC ranked by significance analysis of microarrays (SAM)

**Order**	**Probeset**	**Gene ID**	**Score (*d*)**	**Numerator (*r*)**	**Denominator (*s*+*s*0)**	**Fold change**	***q*-value (%)**
1	39470_at	39470_at	2.5193455	3.596293949	1.427471523	13.48750	5.9170776
2	41470_at	PROM1	2.1986583	3.825972532	1.740139698	10.60748	5.9170776
3	41354_at	STC1	2.0805893	2.751729734	1.322572262	7.74726	5.9170776
4	38207_at	38207_at	2.0726086	3.311991516	1.597982165	6.51440	5.9170776
5	1802_s_at	ERBB2	1.9669529	3.492165691	1.775419064	17.65875	5.9170776
6	39506_at	39506_at	1.9191495	3.359977858	1.750763964	4.15946	5.9170776
7	33218_at	ERBB2	1.8831385	2.592838393	1.376870795	9.12831	5.9170776
8	37978_at	QPRT	1.8508847	2.471411028	1.335259314	3.80801	5.9170776
9	35912_at	MUC4	1.8095618	2.831603133	1.564800451	3.85299	5.9170776
10	41376_i_at	UGT2B7	1.8010151	3.20113721	1.777407186	4.20987	5.9170776
11	37208_at	PSPHL	1.784549	3.621295275	2.029249576	3.44056	5.9170776
12	38545_at	INHBB	1.7806599	3.920162398	2.201522201	4.16889	5.9170776
13	33576_at	KIAA0918	1.7555623	2.180726438	1.242181175	5.31405	5.9170776
14	32521_at	SFRP1	1.7432391	2.824069509	1.620012708	10.60682	5.9170776
15	35704_at	HRASLS3	1.7261492	2.548006268	1.476121705	4.49637	5.9170776
16	33358_at	ARHCL1	1.6665422	1.95881684	1.175377897	3.16918	5.9170776
17	31732_at	RLN2	1.6347725	2.459163154	1.50428461	3.67860	5.9170776
18	38267_at	SLC1A1	1.6306308	2.219928654	1.361392538	4.39416	5.9170776
19	37883_i_at	AF038169	1.6147363	2.219700503	1.374652015	4.98971	5.9170776
20	32963_s_at	RRAGD	1.6121619	1.626523265	1.008908118	3.26944	5.9170776
21	994_at	PTPRM	1.6097261	2.784507191	1.729801847	3.63111	5.9170776
22	995_g_at	PTPRM	1.6088285	2.897830612	1.801205444	3.40453	5.9170776
23	311_s_at	311_s_at	1.5938406	4.139143468	2.596961926	3.01704	5.9170776
24	38268_at	SLC1A1	1.5621501	2.110626126	1.351103322	5.35166	5.9170776
25	31892_at	PTPRM	1.5119824	3.112003216	2.058227135	2.74704	5.9170776
26	35148_at	TJP3	1.510845	1.998741079	1.322929233	2.73928	5.9170776
27	41700_at	F2R	1.5014465	2.195953257	1.462558397	7.18646	5.9170776
28	35674_at	PADI2	1.479696	2.264084328	1.53010105	3.01842	5.9170776
29	1680_at	GRB7	1.4698173	2.027019267	1.379096066	4.25537	5.9170776
30	37209_g_at	PSPHL	1.4664685	1.930835879	1.316656891	2.66282	5.9170776
31	39966_at	CSPG5	1.4455697	1.818363955	1.257887393	3.93459	5.9170776
32	36869_at	PAX8	1.441286	2.813586886	1.95213647	2.99423	5.9170776
33	36202_at	PKIA	1.4322379	2.002737974	1.398327765	3.74274	5.9170776
34	828_at	PTGER2	1.4285229	1.890660776	1.32350749	4.10149	5.9170776
35	39075_at	NEU1	1.4246097	1.46915449	1.031268071	2.50012	5.9170776
36	36990_at	UCHL1	1.4234749	1.966718653	1.381632101	2.81907	5.9170776
37	36943_r_at	PLAGL1	1.4016117	1.503433722	1.072646422	2.57234	5.9170776
38	40488_at	DMD	1.3944472	1.630344124	1.16916874	2.42639	5.9170776
39	35985_at	PALM2	1.3805351	2.00686626	1.4536872	3.22031	5.9170776
40	36254_at	TAC1	1.3634058	2.750616522	2.017459875	6.36232	5.9170776
41	37869_at	ELKS	1.3454118	1.248133462	0.927696233	2.33294	5.9170776
42	2021_s_at	CCNE1	1.3294844	1.454719587	1.094198324	3.06396	5.9170776
43	33878_at	FLJ13612	1.3274937	1.237854188	0.932474615	2.29949	5.9170776
44	39757_at	SDC2	1.3043638	2.072350471	1.588782604	2.35737	5.9170776
45	36508_at	GPC4	1.2991039	1.843342415	1.41893382	2.86605	5.9170776
46	933_f_at	ZNF91	1.2741536	1.376374281	1.080226336	2.37962	5.9170776
47	41269_r_at	API5	1.2739465	1.209800113	0.949647539	2.16666	5.9170776
48	40679_at	SLC6A12	1.2660176	2.282007655	1.802508675	3.78353	5.9170776
49	38168_at	INPP4B	1.2480709	1.359180414	1.089025041	2.54847	5.9170776
50	1860_at	TP53BP2	1.2409357	1.215816901	0.979758188	2.35894	5.9170776
51	38997_at	SLC25A1	1.2115515	1.434841782	1.184301064	2.27730	5.9170776
52	36261_at	LOC51760	1.2106872	1.239580344	1.023865052	2.21646	5.9170776
53	37258_at	TMEFF1	1.1973963	1.515221127	1.265429912	2.66426	5.9170776
54	37884_f_at	AF038169	1.1701381	1.429952351	1.222037264	3.55995	5.9170776
55	39332_at	MGC8685	1.1504101	1.560522625	1.356492456	2.45317	5.9170776
56	40077_at	ACO1	1.1452896	1.324530845	1.15650301	2.29132	5.9170776
57	40194_at	GTF2H2	1.1312711	1.17700447	1.040426546	2.11918	5.9170776
58	36985_at	IDI1	1.1292005	1.378736024	1.220984217	2.55491	5.9170776
59	121_at	PAX8	1.1201623	1.208458366	1.078824359	2.03114	5.9170776
60	977_s_at	CDH1	1.1193705	2.699730529	2.411829333	2.20046	5.9170776
61	35143_at	DKFZP566A1524	1.1183384	1.620344382	1.44888559	2.22206	5.9170776
62	37999_at	CPO	1.1064994	1.06722326	0.964504126	2.06986	5.9170776
63	37210_at	INA	1.1044125	1.281004592	1.159896882	2.40281	5.9170776
64	36175_s_at	HIVEP2	1.0992289	1.174149577	1.068157485	2.19097	5.9170776
65	366_s_at	NEK2	1.0763152	1.152375048	1.070666856	2.16698	5.9170776
66	32893_s_at	GGT2	1.0751984	1.434104513	1.33380457	2.99540	5.9170776
67	41172_at	RDH11	1.0679325	1.000763289	0.937103522	2.07342	5.9170776
68	35978_at	PRRG1	1.0611643	1.103335239	1.039740243	2.04432	5.9170776
69	41715_at	PIK3C2B	1.0489539	0.921860026	0.878837519	2.00231	5.9170776
70	39901_at	EDIL3	1.0487582	1.199399171	1.143637468	2.81306	5.9170776
71	40900_at	MYH10	1.033065	0.971734516	0.940632521	2.13908	6.1516929
72	39436_at	BNIP3L	1.0248207	1.021584326	0.996841974	2.23030	6.1516929
73	32027_at	PDZK1	1.0234011	1.294822852	1.265215452	3.74641	6.1516929
74	1818_at	1818_at	1.0226221	1.159537838	1.13388698	2.17253	6.1516929
75	41644_at	SASH1	1.0075712	0.944905271	0.937804973	1.98700	6.2373918
76	1837_at	1837_at	0.8980795	1.048441576	1.167426238	2.15603	7.6204787
1	37185_at	SERPINB2	−2.712078	−4.21769782	1.555153568	0.04432	5.9170776
2	34439_at	AIM2	−1.887056	−2.3302995	1.234886275	0.14663	17.36191
3	33162_at	INSR	−1.7303429	−2.66873302	1.542314531	0.26385	17.36191
4	40478_at	DJ971N18.2	−1.7156271	−2.71817086	1.584359902	0.13539	17.36191
5	41796_at	PLCL2	−1.6656048	−1.85109255	1.111363586	0.30311	17.36191
6	37816_at	C5	−1.5809083	−2.31648603	1.465288041	0.24600	17.36191
7	859_at	CYP1B1	−1.5772674	−3.24419453	2.056844985	0.20802	17.36191
8	40071_at	CYP1B1	−1.5585486	−3.21119542	2.060375537	0.25050	17.36191
9	39566_at	CHRNA7	−1.5536961	−2.40918109	1.550612809	0.31177	17.36191
10	36073_at	NDN	−1.4829481	−3.50636378	2.364454837	0.14949	17.36191
11	38837_at	DJ971N18.2	−1.4796673	−2.14671946	1.450812223	0.14290	17.36191
12	36077_at	RABL4	−1.4523961	−1.96257543	1.351267383	0.36574	17.36191
13	40395_at	PLXNA2	−1.4373891	−1.79826591	1.251064131	0.29518	17.36191
14	1669_at	WNT5A	−1.4102572	−2.6444399	1.875147235	0.22963	17.36191
15	40387_at	EDG2	−1.3772565	−2.25220008	1.635280028	0.22311	17.36191
16	39805_at	ABCB6	−1.3495984	−2.0539729	1.521914154	0.30094	17.36191
17	32668_at	SSBP2	−1.305583	−1.39323342	1.067135083	0.38204	17.36191
18	38294_at	HOXD4	−1.3021769	−1.80385782	1.385263285	0.26382	17.36191
19	33929_at	GPC1	−1.2952699	−1.49713601	1.155848702	0.32097	17.36191
20	444_g_at	444_g_at	−1.2845736	−1.70421346	1.326676407	0.34439	17.36191
21	1363_at	FGFR2	−1.2806909	−1.57525259	1.230002183	0.24473	17.36191
22	40142_at	DDX24	−1.2701961	−1.27558267	1.004240756	0.46178	17.36191
23	1143_s_at	1143_s_at	−1.2593487	−1.61800179	1.284792503	0.24502	17.36191
24	36453_at	KIAA0711	−1.229961	−1.94610499	1.582249319	0.36118	17.36191
25	514_at	CBLB	−1.2010635	−1.19235629	0.992750384	0.39810	17.36191
26	34853_at	FLRT2	−1.2008015	−1.40750914	1.172141399	0.29102	17.36191
27	40112_at	IDH3B	−1.1932078	−1.31001149	1.097890455	0.43852	17.36191
28	38271_at	MGC16025	−1.1909435	−1.56930168	1.31769611	0.44320	17.36191
29	32381_at	RORB	−1.1900513	−1.68115724	1.412676329	0.44795	17.36191
30	39709_at	SEPW1	−1.1672019	−1.17314959	1.005095698	0.49329	17.36191
31	38042_at	G6PD	−1.1666541	−1.3074933	1.12072066	0.35407	17.36191
32	32610_at	RIL	−1.1500988	−1.87581032	1.630999328	0.38759	17.36191
33	1325_at	MADH1	−1.1063114	−1.23201185	1.113621238	0.44054	17.36191
34	32800_at	RXRA	−1.0647524	−1.19695561	1.124163351	0.46285	17.36191
35	34354_at	FGFR2	−1.0353338	−1.34091	1.295147453	0.33456	17.36191
36	37280_at	MADH1	−1.03448	−1.06866713	1.033047673	0.48063	17.36191
37	33227_at	IL10RB	−1.017976	−1.10172876	1.08227379	0.47487	17.36191
38	32529_at	CKAP4	−0.9990762	−1.12343355	1.124472307	0.48333	17.36191
39	36312_at	SERPINB8	−0.9653125	−1.26934824	1.31496097	0.47187	17.36191
40	1403_s_at	CCL5	−0.9613302	−1.420866	1.478020843	0.30353	17.36191

, Figure 2). OSPC2, the only serous tumour with mixed clear cell histology evaluated in our series, was also found to highly overexpress *c-erbB2* (data not shown).

### Validation of the microarray data

**Figure 3:**
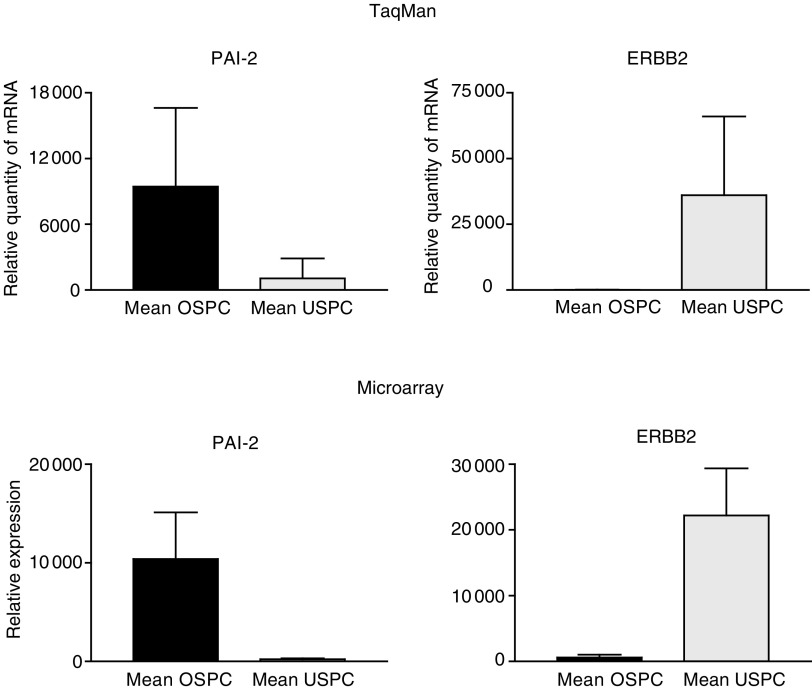
Quantitative RT – PCR and microarray expression analysis of PAI-2 (SERPINB2) and c-erbB2 (ERBB2) selected genes differentially expressed between OSPC and USPC.

We used q-RT – PCR assays to validate the microarray data. The two most highly differentially expressed genes between OSPC and USPC (i.e., *PAI-2* and *c-erbB2*) were selected for q-RT – PCR analysis. A comparison of the microarray and q-RT – PCR data for these genes is shown in Figure 3

. Expression differences between tumour types for *PAI-2* (*P*=0.009) and *c-erbB2* (*P*=0.02), were readily apparent (Tables 2 and 3). Moreover, for both genes tested, the q-RT – PCR data were highly correlated to the microarray data (*P*<0.001) (*r*=0.91 and 0.71, respectively), as estimated from the 6 samples (i.e., three OSPC and three USPC) included in both the q-RT – PCR and microarray experiments. The q-RT – PCR data mirror the microarray data, both qualitatively and quantitatively, and suggest that most array probe sets are likely to accurately measure the levels of the intended transcript within a complex mixture of transcripts.

### HER-2/neu expression

**Figure 4:**
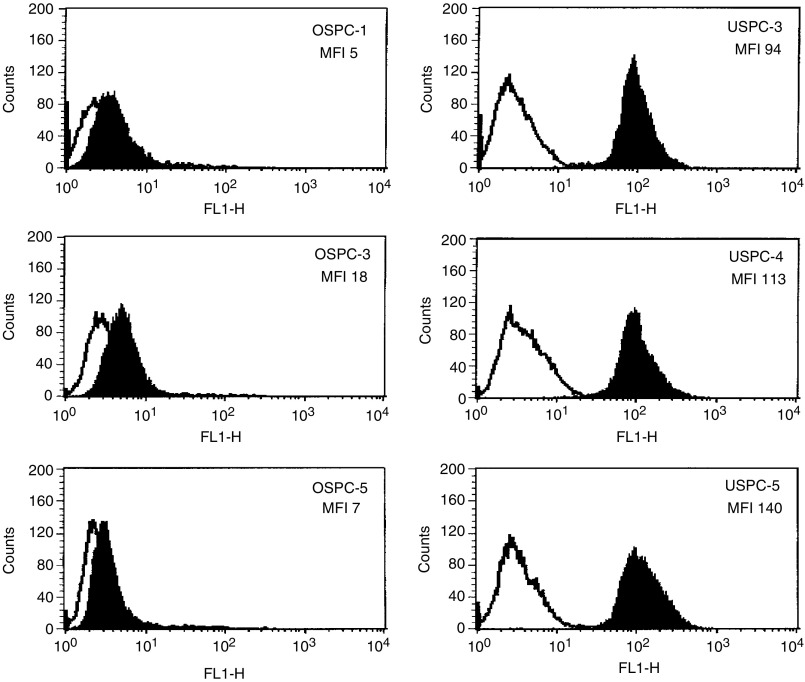
FACS analysis of Herceptin staining of three primary OSPC and three USPC cell lines. Data with Herceptin are shown in solid black while isotype control MAb profiles are shown in white. HER-2/neu expression was significantly higher on USPC cell lines compared to OSPC cell lines (*P*<0.001 by Student's *t-*test).

We evaluated HER-2/neu expression by flow cytometry on six primary serous papillary cell lines (three OSPC and three USPC). As positive and negative controls, breast cancer cell lines known to overexpress HER-2/neu (BT-474 and SK-BR-3, American Type Culture Collection), and Epstein – Barr virus-transformed lymphoblastoid cell lines (LCL) established from the same USPC and OSPC patients were also studied. High HER-2/neu receptor expression was found on all three primary USPC cell lines tested (100% positive cells for all three USPC), with mean fluorescence intensity (MFI) ranging from 94 to 140 (Figure 4

). In contrast, primary OSPC cell lines were found to express significantly lower levels of HER-2/neu (average MFI was 10-fold lower) than the USPC cells (*P*<0.001) (Figure 4). These results show that high expression of the *c-erbB2* gene product by USPC correlates tightly with high protein expression by the tumour cells. Autologous LCL were consistently negative for HER-2/neu expression, while breast cancer cell lines expressed high levels of HER-2/neu (data not shown).

### Immunohistochemical analysis of HER2/neu expression

**Figure 5:**
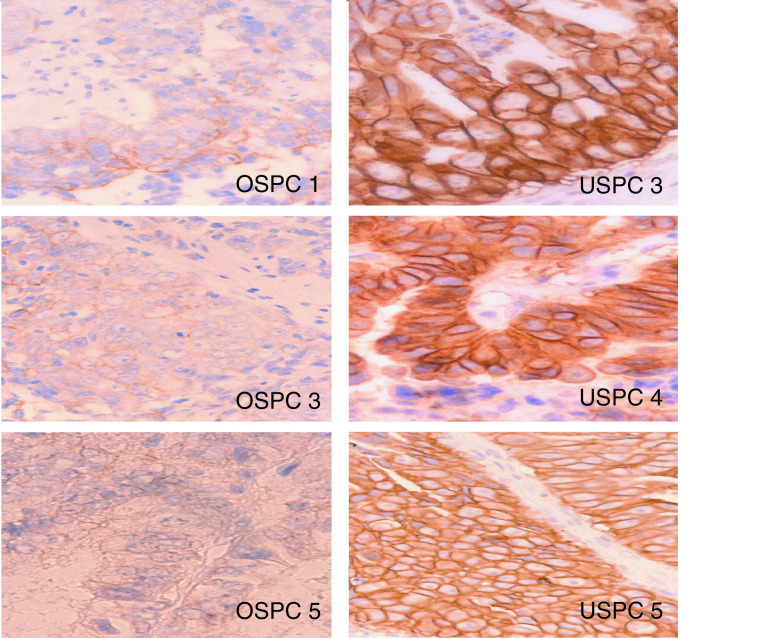
Immunohistochemical staining for HER-2/neu expression on three paraffin-embedded OSPC3 and three USPC5 specimens from which primary cell lines have been established. OSPC1, OSPC3 and OSPC5 (left panel) showed negative or light (1+) staining for HER-2/neu. USPC3, USPC4 and USPC5 (right panel), showed heavy (3+) staining for HER-2/neu. Original magnification × 400.

Formalin-fixed tumour tissue blocks from six primary surgical specimens were tested for HER-2/neu expression. Heavy staining for HER-2/neu protein expression (i.e., score 3+) was noted in all three USPC specimens that also overexpressed the *c-erbB2* gene product by microarray and flow cytometry, respectively (Figure 5

). In contrast, negative or low (i.e., score 0 or 1+) staining was found in all three representative OSPC samples (Figure 5). Similarly, when formalin-fixed tumour tissue blocks from 20 independent surgical specimens (i.e., 10 OSPC *vs* 10 USPC) were tested for HER-2/neu expression, a moderate to heavy staining was found in 70% of USPC (i.e., 70% score 2+ and 3+, 30% score 1+) *vs* 10% of OSPC (i.e., 10% score 2+ and 90% score 0 to 1+) (*P*=0.0002 USPC *vs* OSPC by student's *t*-test).

## DISCUSSION

High-throughput comprehensive technologies for assaying gene expression, such as high-density oligonucleotide and cDNA microarrays, may offer the potential to identify clinically relevant subsets of tumours difficult to distinguish by conventional histopathological assessment (Giordano *et al*, 2001; Rosenwald *et al*, 2002; Schwartz *et al*, 2002). This report represents the first communication of an investigation involving the genome-wide examination of differences in gene expression between serous papillary ovarian cancer (OSPC) and uterine serous papillary carcinoma (USPC), two histologically indistinguishable gynaecologic tumours characterised by a dramatically different biologic behavior and response to chemotherapy.

Advanced and/or metastatic serous papillary gynaecologic tumours, regardless of their ovarian or uterine origin, are currently treated with a combined cisplatinum-based chemotherapy. However, given that: (1) USPC likely arise from metaplastic Mullerian epithelium, while OSPC likely derive from the ovarian surface epithelium, and (2) a dramatic difference in response to standard chemotherapy regimens is commonly reported among these histologically indistinguishable serous carcinomas (Levenback *et al*, 1992; Sherman *et al*, 1992; Carcangiu and Chambers, 1995; Nicklin and Copeland, 1996; Kalil and McGuire 2002), a significant diversity in gene expression among these tumours is probable. In agreement with this view, all five USPC patients evaluated in this study either developed progressive disease during chemotherapy or recurred within 6 months from the end of treatment. In contrast, four out of five of the OSPC patients responded completely to standard adjuvant chemotherapy treatment. In this study, we have used short-term primary OSPC and USPC cultures (to minimise the risk of a selection bias inherent in any long-term *in vitro* growth) to study differential gene expression in highly enriched populations of epithelial tumour cells. Strikingly, we found that hierarchical clustering of the samples and gene expression levels within the samples led to the unambiguous separation of OSPC from USPC. We detected 116 genes differentially expressed between OSPC and USPC whose average change in expression level between the two groups was at least two-fold. Of the 116 genes that yielded *P*<0.05 via WRS, all 116 were among the genes found significant by SAM. Our study offers therefore the first persuasive support that the dramatically different biologic behaviour and response to treatment commonly reported in OSPC compared to USPC may be dictated by a profound genetic diversity among these histological indistinguishable serous neoplasms. It is therefore likely that a molecular classification based on gene expression profiles may thus potentially identify gynaecologic serous tumours associated with aggressive behaviour and poor prognosis and should allow therapeutic approaches to be better tailored to the biologic and genetic characteristic of each serous tumour type. These novel findings have thus the potential to significantly refine diagnosis and possibly alter management of these cancer patients. Of interest, OSPC2, the only OSPC with mixed clear cell features included in our analysis, clustered with USPC. These data are congruent with a recent report that clear cell ovarian tumours present a distinctive molecular signature from pure high-grade OSPC (Schwartz *et al*, 2002). Thus, our findings add to previous knowledge showing that clear cell tumours, a variant of ovarian cancer with a particularly unfavourable prognosis, express a molecular signature closer to that of the more aggressive USPC.

A sizeable number of genes differentially expressed in OSPC compared with USPC have been identified through our analysis. Some of these may prove to be useful diagnostic and therapeutic markers for these histologically similar diseases. For example, elevated serum levels of lysophosphatidic acid (LPA) are found in more than 90% of ovarian cancer patients and the level of LPA in plasma has been proposed as a potential biomarker for this disease (Budnik and Mukhopadhyay, 2002). In addition, LPA signalling may have a role in the progression of ovarian cancer cells through stimulation of cellular proliferation, enhanced cellular survival and suppression of apoptosis (Contos *et al*, 2000). It seems therefore likely that the higher LPA receptor expression found in OSPC relative to USPC may represent a distinctive marker that plays a role in transduction of growth-promoting signals from high local concentrations of LPA (Contos *et al*, 2000; Budnik and Mukhopadhyay, 2002). Consistent with this view, phospholipase C, another gene that is differentially overexpressed in OSPC relative to USPC has been previously reported to contribute to LPA production in ovarian cancer cells (Budnik and Mukhopadhyay, 2002).

Several reports have shown that plasminogen activator inhibitor-2 (PAI-2), a protein capable of inhibiting invasion (Andreasen *et al*, 2000), may represent a molecular biomarker for several human tumours including ovarian carcinomas. Consistent with our findings, however, overexpression of PAI-2 in epithelial ovarian cancer has been previously identified as a favourable prognostic factor (Chambers *et al*, 1997). Indeed, high PAI-2 expression in invasive ovarian tumours seem to be limited to a group of OSPC patients which experience a more prolonged disease free and overall survival (Chambers *et al*, 1997). These data are therefore consistent with the view that high expression of PAI-2 in OSPC compared to USPC may be a marker indicating a biologically less aggressive disease.

Membrane-associated heparan sulphate proteoglycans are thought to play important roles in many aspects of cell behaviour, including cell – cell and cell – extracellular matrix adhesion and growth factor signalling (David, 1993). Two families of polypeptides appear to carry the majority of heparan sulphate on mammalian cells: glypicans, which are attached to the plasma membrane via glycosylphosphatidylinositol (GPI) anchors, and syndecans, which are transmembrane proteins (David, 1993). Convincing evidence has recently been provided that glypican-1 can interact with FGF-2 and stimulate signalling of the FGF receptor (Steinfeld *et al*, 1996). Importantly, high glypican-1 and FGF receptor 2 gene expression were found differentially expressed in OSPC when compared to USPC, while syndecan-2 gene expression was significantly higher in USPC when compared USPC. These data therefore support a major difference in the expression of heparan sulphate proteoglycans between these two subsets of histologically indistinguishable serous tumours. Furthermore, because bFGF is produced by OSPC and can bind to FGF receptor 2 expressed on these tumours (Steinfeld *et al*, 1996), it is likely that the combined overexpression of glypicans and FGF receptor 2 genes found in OSPC may represent a common molecular abnormality with important functional consequences for the progression of OSPC.

Insulin receptor has been previously reported overexpressed on OSPC and to be able to mediate a proliferative response in ovarian cancer cells (Kalli *et al*, 2002). In our study, consistent with previous reports, OSPC were found to differentially overexpress the insulin receptor gene when compared to USPC. These results therefore support a role for insulin receptor in the growth and regulation of OSPC, but not in USPC.

Unlike OSPC, there have been remarkably few studies aimed at identifying molecular markers characteristic of USPC. Because of the common poor response to standard salvage treatment modalities for advanced or recurrent USPC, the identification of a number of USPC specific markers may lay the groundwork for future studies testing some of these biomarkers for clinical utility in the treatment of these highly aggressive and intrinsically chemotherapy resistant tumours. Of great interest at this regard, c-erbB2 gene was found to be the most highly differentially expressed gene in USPC with over 17-fold upregulation compared with OSPC. Furthermore, the growth factor receptor-bound protein 7 (GRB7), a gene tightly linked to c-erbB2 and previously reported coamplified and coexpressed with this gene in several cancer types (Janes *et al*, 1997) was also highly differentially expressed in USPC compared to OSPC. The striking overexpression of the c-erbB2 gene as well as of its gene expression product on USPC may therefore represent a distinctive molecular marker for these serous tumours and also provide insights into the disproportionately poor prognosis of USPC relative to OSPC. Consistent with this view, previous studies have reported that the amplification of this gene in a subset of ovarian cancer patients is associated with resistance to chemotherapeutic drugs and shorter survival (Berchuck *et al*, 1990). On the light of our micrarrays data it is tempting to speculate that some if not all of these highly HER2/neu overexpressing and chemotherapy resistant serous tumours may likely have arisen from metaplastic mesothelial cells and therefore present a genetic fingerprint more similar to USPC than OSPC. Regardless of the histologic site of origin, however, high overexpression of the c-erbB2 gene provides support for the notion that trastuzumab (Herceptin), a humanised anti-HER-2/Neu antibody that is showing great promise for treatment of metastatic breast cancer patients overexpressing HER-2/Neu protein (Slamon *et al*, 2001), may be a novel, potentially highly effective therapy against this aggressive variant of serous papillary carcinomas. Consistent with this view, our group has recently shown high sensitivity of USPC to the killing activity mediated by natural killer (NK) cells when triggered by anti-HER-2/Neu-specific antibody *in vitro* (Santin *et al*, 2002b).

Taken all together, our data demonstrate that OSPC and USPC, two diseases where further molecular characterisation is needed to improve differential diagnosis and therapeutic strategies, can be readily discriminated solely by gene expression profiles. These findings suggest that global gene expression signatures can be an important adjunct to the morphology based classification schemes for serous papillary tumours currently used. Finally, the identification of c-erbB2 as the most highly differentially expressed gene in USPC suggest that targeting HER-2/neu by rhuMAb anti-HER-2 (Herceptin) may be potentially highly beneficial against these biologically aggressive and chemotherapy-resistant variants of endometrial cancer.
